# Comparative Performance of Multivariable Agro-Physiological Parameters for Detecting Salt Tolerance of Wheat Cultivars under Simulated Saline Field Growing Conditions

**DOI:** 10.3389/fpls.2017.00435

**Published:** 2017-03-29

**Authors:** Salah E. El-Hendawy, Wael M. Hassan, Nasser A. Al-Suhaibani, Yahya Refay, Kamel A. Abdella

**Affiliations:** ^1^Department of Plant Production, College of Food and Agriculture Sciences, King Saud UniversityRiyadh, Saudi Arabia; ^2^Department of Agronomy, Faculty of Agriculture, Suez Canal UniversityIsmailia, Egypt; ^3^Department of Agricultural Botany, Faculty of Agriculture, Suez Canal UniversityIsmailia, Egypt; ^4^Department of Biology, College of Science and Humanities at Quwayiah, Shaqra UniversityRiyadh, Saudi Arabia

**Keywords:** chlorophyll fluorescence, ion contents, photosynthesis, PCA, subsurface water retention technique, *Triticum aestivum*, water relations

## Abstract

Field-based trials are crucial for successfully achieving the goals of plant breeding programs aiming to screen and improve the salt tolerance of crop genotypes. In this study, simulated saline field growing conditions were designed using the subsurface water retention technique (SWRT) and three saline irrigation levels (control, 60, and 120 mM NaCl) to accurately appraise the suitability of a set of agro-physiological parameters including shoot biomass, grain yield, leaf water relations, gas exchange, chlorophyll fluorescence, and ion accumulation as screening criteria to establish the salt tolerance of the salt-tolerant (Sakha 93) and salt-sensitive (Sakha 61) wheat cultivars. Shoot dry weight and grain yield per hectare were substantially reduced by salinity, but the reduction was more pronounced in Sakha 61 than in Sakha 93. Increasing salinity stress caused a significant decrease in the net photosynthesis rate and stomatal conductance of both cultivars, although their leaf turgor pressure increased. The accumulation of toxic ions (Na^+^ and Cl^-^) was higher in Sakha 61, but the accumulation of essential cations (K^+^ and Ca^2+^) was higher in Sakha 93, which could be the reason for the observed maintenance of the higher leaf turgor of both cultivars in the salt treatments. The maximum quantum PSII photochemical efficiency (*F*_v_/*F*_m_) and the PSII quantum yield (ΦPSII) decreased with increasing salinity levels in Sakha 61, but they only started to decline at the moderate salinity condition in Sakha 93. The principle component analysis successfully identified the interrelationships between all parameters. The parameters of leaf water relations and toxic ion concentrations were significantly related to each other and could identify Sakha 61 at mild and moderate salinity levels, and, to a lesser extent, Sakha 93 at the moderate salinity level. Both cultivars under the control treatment and Sakha 93 at the mild salinity level were identified by most of the other parameters. The variability in the angle between the vectors of parameters explained which parameters could be used as individual, interchangeable, or supplementary screening criteria for evaluating wheat salt tolerance under simulated field conditions.

## Introduction

Over 20% of the irrigated land and more than 6% of the world’s total land are now within the ambit of the salt effects (Mickelbart et al., 2015). In addition, about 1.5 Mha of arable land is lost and $27.5 billion is spent annually due to the salinity problem in the agricultural sector (FAO, 2010; Qadir et al., 2014). Furthermore, water scarcity in arid and semiarid regions, where more than 40% of the world population resides, is leading toward an increase in the amount of saline or brackish water used for irrigating essential food crops, such as wheat. All of these facts about salinity suggest that it is one of the most severe environmental stresses affecting human life.

Wheat (*Triticum aestivum* L.) is only moderately tolerant to salinity; the loss in its grain yield exceeds 60% due to soil salinity (El-Hendawy et al., 2009; Saleem et al., 2011). There are several agronomic management practices that can alleviate the adverse effects of salinity stress on the growth and yield of wheat: for example, the mixing of large quantities of gypsum into the soil, and the use of effective drainage schemes and leaching portion. However, these practices are still prohibitively expensive, provide only a short-term solution, and are not feasible to apply on large scales. Furthermore, most farmers do not have the required skills and sufficient water to apply them. Therefore, one of the most effective and feasible ways to sustain wheat production under high salinity conditions is to enhance the tolerance of wheat genotypes to salt stress (El-Hendawy et al., 2005b; Munns, 2008). Unfortunately, success in developing salt-tolerant wheat genotypes has remained limited so far. This is due to several factors, including but not limited to: (1) the lack of understanding of salt tolerance mechanisms; (2) the evaluation of salt tolerance being focused on the grain yield criterion, which reflects tolerance to salinity at the whole plant level only; (3) the majority of salinity experiments being done under tightly controlled conditions, using solutions and sand culture, which fail to express the complexity of saline soils that affect the soil–plant interactions under field conditions; and (4) the relatively infrequent use of physiological characteristics as selection criteria for salt tolerance, which can reflect the response to salt stress at the organ, tissue, and cellular levels (El-Hendawy et al., 2005a,b; Genc et al., 2010; Tavakkoli et al., 2010).

The prerequisite for improving salt tolerance of genotypes in breeding programs is the identification of the agro-physiological parameters that have potential as screening criteria for discriminating wheat genotypes for salt tolerance. Most importantly, it is difficult to accept any quantitative parameters as screening criteria without testing them first under natural field conditions, where the plants are exposed to temporal and spatial variation in salt concentration and water content in the root zone at different growth stages, and experience high variability in temperature and humidity that control the evapotranspiration rate. On the other hand, these natural field characteristics make the identification of potential quantitative parameters, especially physiological parameters, as screening criteria very difficult. Therefore, in this study, we tested the comparative performance of multivariable agro-physiological parameters as screening criteria for evaluating the salt tolerance of wheat cultivars using the subsurface water retention technique (SWRT). This technique simulates close-to-field conditions through providing a larger measuring area and sufficient sample sizes, and creating temporal and spatial variation in salt concentration and water content in the root zone at different growth stages. In addition, this technique permits control of the amount and frequency of saline irrigation water that is applied during the growing seasons. For SWRT, polyethylene (PE) membranes are installed beneath the root zones, about 40 cm below the soil surface. The installation of the membranes is described in detail in the Section “Materials and Methods.”

Excess of salt concentrations in the root zone causes substantial changes in various morphological and physiological traits at various organizational levels in the plant (whole plant, tissue and cellular levels). This is achieved through osmotic stress, specific ion toxicities, and ion imbalance (Ashraf and Harris, 2004; Parida and Das, 2005). High salt concentrations in the root zone result in an increased osmotic stress in the soil solution and a consequent decrease in water availability to the plant, in a similar manner to drought stress. Specific ion toxicities, which become visible over prolonged periods, correspond to the excessive buildup of Na^+^ and Cl^-^ in the leaf blade, reaching toxic levels (Nawaz et al., 2010). The antagonism that exists between Na^+^ and essential cations, particularly K^+^ and Ca^2+^, in the site of ion uptake in the roots, causes ion imbalance at cellular and tissue levels by reducing the ratios of K^+^/Na^+^ and Ca^2+^/Na^+^ (Elhamid et al., 2014; De Leon et al., 2015). Salt-tolerant genotypes can overcome the negative impacts of these events by generating distinct salt tolerance mechanisms. Since various morphological and physiological characteristics can contribute to salt tolerance mechanisms, several studies have demonstrated that using the specific morphological and physiological traits underlying salt tolerance mechanisms as screening criteria will contribute to making the evaluation of salt tolerance among genotypes more effective (El-Hendawy et al., 2005a,b; Munns and Tester, 2008; Nawaz et al., 2010; Zhang et al., 2014; Ashraf and Ashraf, 2015).

El-Hendawy et al. (2005a) reported that the substantial reduction in the growth of salt tolerant wheat genotypes under salinity stress was primarily related to a decline in photosynthetic capacity, rather than a reduction in leaf area. Zarco-Tejada et al. (2003) also mentioned that the negative impact of salinity stress on the photosynthetic apparatus, especially photosystem II (PSII), could be detected before the irreversible morphological damage is visible. Moreover, the buildup of Na^+^ and Cl^-^ in the leaf blade to toxic concentrations is known to reduce the photosynthesis rate through the destruction of chlorophyll ultrastructures and an inhibition of PSII at both donor and acceptor sides (Chen and Murata, 2011). Furthermore, any environmental stress that has a negative effect on the operating efficiency of PSII was shown to have an effect on chlorophyll fluorescence (Naumann et al., 2008). Zhang et al. (2011) also mentioned that the early detection of chlorophyll fluorescence could be used as an indicator to avoid loss of plant biomass under high salinity conditions. This close relationship between salinity stress and photosynthesis efficiency allows us to use all parameters related to the photosynthetic apparatus as useful screening criteria for distinguishing salt-tolerant genotypes from salt-sensitive ones.

The adaptation of genotypes to ion imbalance in the root zone under high salt concentrations cannot be ruled out as a mechanism of salt tolerance. Therefore, all parameters related to the accumulation and the resulting ratios of ions, particularly Na^+^, K^+^, and Ca^2+^, could be considered as key physiological criteria for the differentiation between salt-tolerant and salt-sensitive genotypes. In most salinity studies with various crops, the salt tolerance has been found to be related to the potential of genotypes to restrict Na^+^ influx or compartmentalize it into the vacuole, as well as permanent acquisition of K^+^ and Ca^2+^ (El-Hendawy et al., 2005a; Nawaz et al., 2010; Zhang et al., 2014; Ashraf and Ashraf, 2015; Oyiga et al., 2016).

Plant growth inhibition under high salinity conditions is related not only to disturbances and imbalances of ions in the soil solution, but also to poor water relations. High salt concentrations in the soil solution depress the soil water potential, which subsequently results in an increased leaf water potential. Therefore, the success of genotypes in adapting to low soil water potential is associated with their ability to lower the leaf water potential sufficiently to increase water uptake. The genotypes can reduce their water potential by decreasing the leaf osmotic potential, either through the accumulation of inorganic ions, or through the synthesis of organic osmolytes (Hasegawa et al., 2000; Nawaz et al., 2010; Singh et al., 2010). Active accumulation of osmotic adjustment components under salt stress helps the plant to maintain higher leaf turgor, which is one of the main mechanisms for ensuring water uptake and enhancing plant growth under salinity conditions (Pérez-López et al., 2009; Ashraf and Ashraf, 2015). Therefore, the salt tolerance of genotypes can be efficiently evaluated using leaf water relations parameters due to the close correlation between salt concentrations in soil solutions and plant water relations parameters.

The objective of this study was to examine the efficiency of multivariable agro-physiological parameters, including leaf water relations, the photosynthetic efficiency apparatus, and ion concentrations as screening criteria to distinguish salt-tolerant wheat cultivars from salt-sensitive ones. The other objective was to reveal the relationships between all tested parameters and identify which parameters could be employed as reliable screening criteria for selection and the improvement of salt tolerance. The collected data will not only be important for testing the suitability of the aforementioned physiological parameters as screening criteria, but also for understanding the mechanisms of salt tolerance in wheat, specifically under simulated field conditions.

## Materials and Methods

### Plant Materials, Experimental Site, and Growth Conditions

Two different wheat cultivars, Sakha 61 and Sakha 93, were used in this study. Both cultivars have been evaluated previously in different screening experiments and the results of those experiments confirmed that Sakha 61 and Sakha 93 are salt-sensitive and salt-tolerant cultivars, respectively (El-Hendawy et al., 2005a,b; Hackl et al., 2013).

The two cultivars were grown in a close-to-field platform using the SWRT at the Experimental Research Station of the College of Food and Agriculture Sciences, King Saud University, Riyadh, Saudi Arabia during the 2014/2015 and 2015/2016 growing seasons. The weather conditions during the entire wheat growth period were in the range of 8 – 25 mm and 12.9 – 32.2°C for rainfall and temperature, respectively. The texture of the experimental soil is sandy throughout its profile (90.4% sand, 5.4% silt and 4.2% clay), with a soil bulk density of about 1.48 g cm^-3^, a field capacity of 0.101 m^3^ m^-3^, and a wilting point of 0.038 m^3^ m^-3^. The soil bulk density was determined according to Grossmann and Reinsch (2002), whereas the water content at field capacity and wilting point were determined using the pressure plate technique as described by Klute (1986).

### Experimental Design and Salinity Treatments

The field experiment was replicated three times and conducted in a randomized complete block split-plot design, with the three levels of salinity and the two cultivars were assigned as the main plots and the subplots, respectively. Each cultivar was sown at a seeding rate of 14 g m^-2^ in a four-row plot, with a plot size 6 m × 0.6 m. The plants were fertilized with 12.0 and 12.3 g m^-2^ of N and P, respectively. Nitrogen fertilizer was applied in three equal doses at seeding, stem-elongation, and booting stages as ammonium nitrate, whereas the whole amount of phosphorus was applied basally before sowing as monocalcium phosphate.

Three water irrigation salinity levels were used in this study: control (0.35 dS m^-1^), mild (6.0 dS m^-1^), and moderate (12.0 dS m^-1^). To avoid osmotic shock during germination and at the early seedling stage, all plots were first irrigated with fresh water for 25 days; thereafter, mild and moderate salinity plots were irrigated with artificial saline water containing 3.51 and 7.02 g NaCl L^-1^, respectively. The control plots continued to be irrigated with fresh water. To ensure the salinity levels of each treatment, the electrical conductivity (EC) of the artificial saline water was measured at each irrigation. A surface irrigation system was used. The main irrigation line, which delivered water from plastic water storage tanks (3.0 m^3^) to the treatment plots, was distributed to sub-main hoses at each plot and equipped with manual control valves in order to deliver equal and constant amounts of water to each plot. The next irrigation was initiated when a 50% or below of the total available water in the root zone was depleted. Soil water content at depths of 0 – 40 cm was used to calculate the quantity of irrigation water for each treatment using the following equation:

$$\text{SMD}=(\theta_{\text{FC}}-\theta_{\text{i}})\times \text{D}\times \text{BD}$$

Where SMD is the soil moisture deficit (mm); θ_FC_ and θ_i_ are the volumetric soil water content (m^-3^ m^-3^) at field capacity and before irrigation, respectively; D is the depth of root (40 cm); and BD is the bulk density of the soil layer (g cm^-3^).

Just before each irrigation, soil samples at depths of 0 – 40 cm were collected from all plots in order to monitor built up of salt concentrations in the root zone and during the growing season at prescribed salinity levels. The EC of soil samples was measured using the soil water extract method, with suspensions with a 2:1 water-to-soil ratio (100 mL of distilled water to 50 g of air-dried soil). Based on the analysis of these samples, the EC of control, mild, and moderate salinity treatments increased from 0.55, 6.1, and 12.2 dS m^-1^ at the tillering stage to 0.57, 8.9, and 14.3 dS m^-1^ at the grain dough stage, respectively.

### Setup of Subsurface Water Retention Technique

The SWRT polyethylene membranes with 3.0 mm thickness were installed at the depth of 40 cm under the soil surface. This depth was chosen according to Fageria and Moreira (2011), who reported that the root biomass of most cereal crops is concentrated at the 0 – 40 cm soil depth. The membranes were installed in a U-shape with a 3:1 width/depth aspect ratio. The right and left sides of the membrane sheet were uplift for 20 cm, whereas the width of the base membrane was kept at 60 cm. In order to allow drainage, a 20 cm wide strip on either side of the membrane sheet was left without a membrane.

### Measurements

#### Growth and Yield Parameters

At the flowering stage, plant dry weight (PDW) was determined for 10 plants collected randomly from each subplot. The harvested samples were dried at 70°C in a forced-air oven for 72 h and weighed to obtain the dry weight per plant. Upon reaching maturity, the two internal rows in each subplot, each 5 m in length, were harvested and threshed to determine the total grain yield per hectare (GYPH). The total grain yield was determined after the weight was adjusted to take into account the seed water content, assumed to be 15%.

#### Water Relations Parameters

The youngest fully expanded and sun-exposed leaves were excised at the flowering stage and used for the measurements of plant water relations parameters: leaf water potential and osmotic potential. Leaf water potential of three leaves from each subplot was measured using a Scholander pressure chamber (Scholander et al., 1965). Leaf osmotic potential was measured after the same leaves used for measuring leaf water potential were frozen in dry ice. Then, the leaf samples were thawed at room temperature and leaf sap was extracted under pressure. The osmotic potential of 10 μL of extracted sap was measured using a vapor pressure osmometer (Wescor 5100C, Wescor Inc., Logan, UT, USA). Leaf turgor potential was recorded as the difference between the values of leaf water potential and leaf osmotic potential.

#### Photosynthetic Parameters

The net photosynthesis rate and stomatal conductance were also measured at the flowering stage in the leaves of three randomly selected plants from each subplot with a portable gas exchange system (Li-6400, Li-COR Inc., Lincoln, NE, USA) at 9:30 – 11:30 AM. Photosynthetic parameters were measured in the second fully expanded leaf from top of each plant.

#### Chlorophyll Fluorescence Parameters

Leaf chlorophyll fluorescence parameters were measured on the same leaves used for measuring photosynthetic parameters using a portable fluorimeter (PAM-2100, Walz, Germany). The selected leaves were adapted to darkness by covering them for 30 min using plastic light exclusion clips. Then, the minimal fluorescence (*F_o_*, with all PSII reaction centers open) was measured using modulated light (<0.1 μmol m^-2^ s^-1^), which was not enough to induce any significant variable fluorescence, and the maximal fluorescence (*F_m_*, with all reaction centers closed) was measured using a 0.8 s saturating light (8000 μmol m^-2^ s^-1^) on a dark-adapted leaf. Subsequently, the same leaves were exposed to actinic light (5000 μmol m^-2^ s^-1^) until a steady-state fluorescence (*F_s_*) was reached and recorded, and a new 0.8 s saturating light at 8000 μmol m^-2^ s^-1^ was applied to determine the maximal fluorescence (*F′_m_*) for light-adapted leaves. The minimal fluorescence (*F′_o_*) in the light-adapted leaves was measured by covering the leaf with a darkening cloth and applying far-red light after switching off actinic light. The *F_o_* and *F_m_* values were used to calculate the variable fluorescence (*F_v_*, calculated as *F_m_* – *F_o_*) and the maximum quantum PSII photochemical efficiency was expressed as the ratio of *F_v_*/*F_m_*. The above fluorescence parameters were also used to calculate the quantum yield of PSII [ΦPSII = (*F*′*_m_* – *F_s_*)/*F*′*_m_*], the photochemical quenching coefficient [qP = (*F*′*_m_* – *F_s_*)/(*F*′*_m_* – *F*′*_o_*)], and the non-photochemical quenching [NPQ = (*F_m_* – *F*′*_m_*)/*F*′*_m_*].

#### Shoot Ion Concentrations

Oven-dried samples of shoots (leaves and stems together) collected at the flowering stage were ground into a fine powder. Approximately 0.4 g of each sample was digested by soaking it in 8 mL concentrated HNO_3_ and 3 mL HClO_4_ for 12 h and then burning at 300°C for 3 h. The digested samples were brought up to a final volume of 50 mL by adding distilled water. The concentrations of Na^+^, K^+^ and Ca^2+^ were then determined using a flame photometer (ELEX 6361, Eppendorf AG, Hamburg, Germany) and, subsequently, the ratios of K^+^/Na^+^ and Ca^2+^/Na^+^ were calculated. For Cl^-^ concentrations, about 0.1 g of the ground-up sample was extracted in 100 mL of deionized water and then shaken for 1 h and filtered (El-Hendawy et al., 2005a). Chloride concentration was determined using an ion chromatography analyzer (Dionex X-300; Sunnyvale, CA 94086, USA).

### Statistical Analysis

Data for all measurements were subjected to ANOVAs appropriate for a randomized complete block split-plot design with salinity level as the main factor and cultivar as the split factor. To test the effects of salinity level, cultivar, and their first- and second-order interactions, the *F*-values of ANOVA test were calculated for all parameters after salinity level, cultivar, and replications considered as random effects using the MIXED procedure. The differences between the mean values were compared using Duncan’s test at 95% probability levels. Data (mean ± standard error of crude data) were presented graphically using Sigmaplot (Sigmaplot for Windows v.12.0, Systat Software Inc., San Jose, CA, USA). In order to obtain a multivariable view of all parameters and two cultivars at different salinity levels, as well as the interrelationships between all parameters, the mean values of all parameters for two cultivars and three salinity levels were subjected to principal component analysis (PCA). The PCA was performed based on correlation matrix data.

## Results

### Growth and Yield Parameters

**FIGURE 1:**
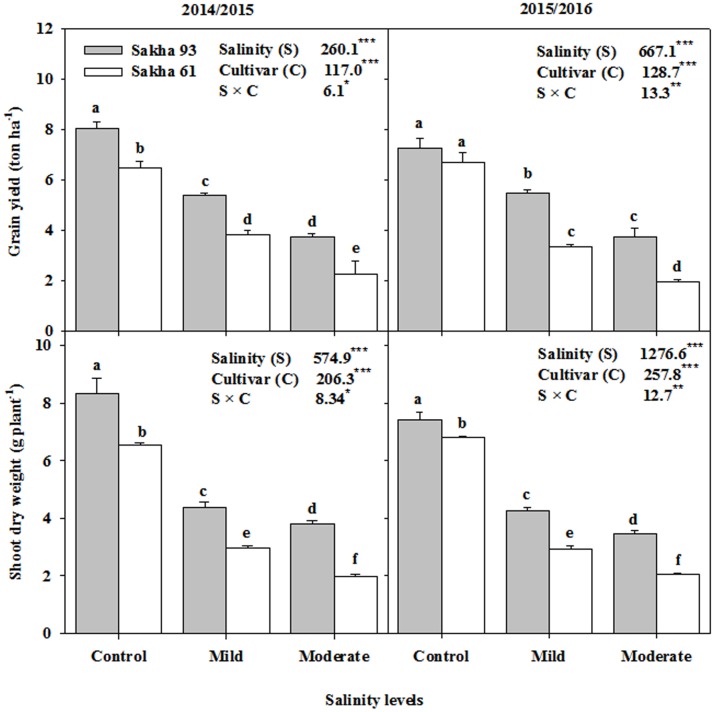
**Effects of different salinity levels on shoot dry weight at flowering stage and grain yield per hectare of two wheat cultivars in 2014/2015 and 2015/2016**. Vertical bars indicate standard error (*n* = 3). Bars labeled with the different letters are significantly different at *P* < 0.05. The stars ^∗^, ^∗∗^ and ^∗∗∗^ indicate significant at 0.05, 0.01, and 0.001, respectively, according to *F*-values of ANOVA test.

Mild and moderate salinity levels led to significantly lower (*P* < 0.001) PDW at the flowering stage and the GYPH than those of the control. Averaged across the two seasons, the percentage reduction reached 50.0 and 61.2% for PDW, and 36.8 and 59.1% for GYPH at mild and moderate salinity levels, respectively (**Figure 1**). However, the reduction in both parameters at both salinity levels from that of the control was lower in the salt-tolerant cultivar Sakha 93 than in the salt-sensitive cultivar Sakha 61. The value of GYPH for Sakha 93 in the moderate salinity treatment was comparable to that exhibited by Sakha 61 in the mild salinity treatment. Significant salinity level and cultivar interactions (S × C) were also observed for both parameters in the two growing seasons (**Figure 1**).

### Water Relations Parameters

**FIGURE 2:**
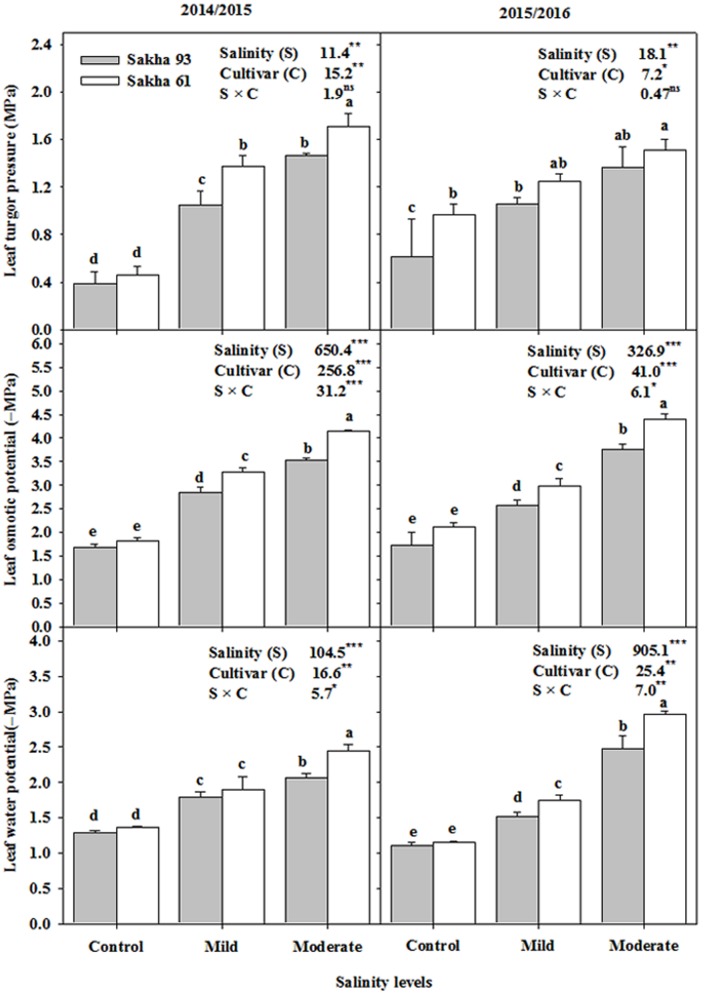
**Effects of different salinity levels on leaf water potential, osmotic potential and turgor pressure at flowering stage of two wheat cultivars in 2014/2015 and 2015/2016**. Vertical bars indicate standard error (*n* = 3). Bars labeled with the different letters are significantly different at *P* < 0.05. The stars ^∗,^^∗∗^ and ^∗∗∗^ indicate significant at 0.05, 0.01, and 0.001, respectively, according to *F*-values of ANOVA test.

As shown in **Figure 2**, the leaf water potential and leaf osmotic potential significantly decreased (became more negative) in both cultivars as salinity increased. However, this reduction in both parameters at mild and moderate salinity levels was greater in the salt-sensitive cultivar Sakha 61 than in the salt-tolerant cultivar Sakha 93 (**Figure 2**). Both salinity levels caused a significant increase in leaf turgor pressure in both cultivars. However, the leaf turgor pressure under salt stress conditions was significantly greater in Sakha 61 than in Sakha 93. Significant S × C interactions were observed for leaf water and osmotic potentials but not for leaf turgor pressure in the two growing seasons (**Figure 2**).

### Photosynthetic Parameters

**FIGURE 3:**
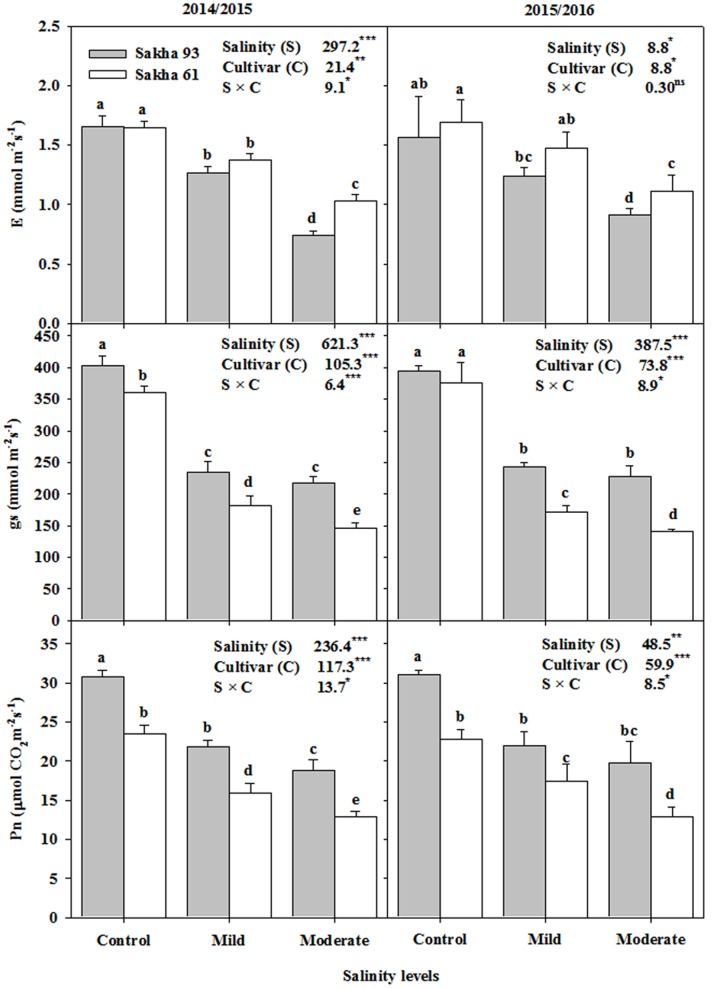
**Effects of different salinity levels on photosynthetic rate (Pn), stomatal conductance (gs) and respiration rate (E) at flowering stage of two wheat cultivars in 2014/2015 and 2015/2016**. Vertical bars indicate standard error (*n* = 3). Bars labeled with the different letters are significantly different at *P* < 0.05. The stars ^∗,^^∗∗^ and ^∗∗∗^ indicate significant at 0.05, 0.01, and 0.001, respectively, according to *F*-values of ANOVA test.

According to *F*-test results, significant differences (*P* < 0.05) between salinity levels, cultivars and their interactions were detected for all photosynthetic parameters [net photosynthesis rate (Pn), stomatal conductance (gs) and respiration rate (E)], except the interaction of salinity and cultivars for E (**Figure 3**). In general, the photosynthetic parameters of both cultivars significantly decreased with increasing salinity levels. However, this decrease was more pronounced in the salt-sensitive cultivar Sakha 61 than in the salt-tolerant cultivar Sakha 93. Interestingly, the differences in Pn and gs between the two cultivars were obvious even in the control treatment. Sakha 93 always exhibited Pn and gs values greater than those of Sakha 61 (**Figure 3**). The difference in E between the two cultivars was obvious and significant only at the moderate salinity level, with Sakha 93 exhibiting a lower E than that of Sakha 61.

### Chlorophyll Fluorescence Parameters

**FIGURE 4:**
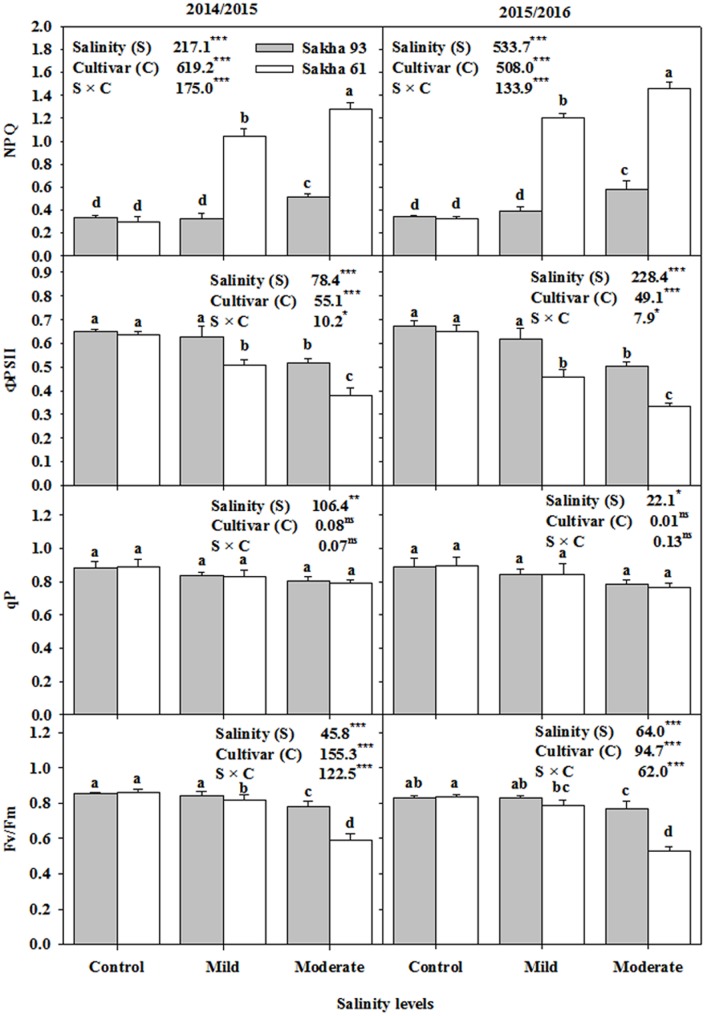
**Effects of different salinity levels on maximum quantum efficiency of PSII photochemical (*F*_v_/*F*_m_), photochemical quenching (qP), quantum yield of PSII (ΦPSII) and non-photochemical quenching (NPQ) at flowering stage of two wheat cultivars in 2014/2015 and 2015/2016**. Vertical bars indicate standard error (*n* = 3). Bars labeled with the different letters are significantly different at *P* < 0.05. The stars ^∗,^^∗∗^ and ^∗∗∗^ indicate significant at 0.05, 0.01, and 0.001, respectively, according to *F*-values of ANOVA test.

Among the four leaf chlorophyll fluorescence parameters, the two cultivars did not differ in photochemical quenching (qP), while showing significant differences in the other three parameters, i.e., the maximum quantum PSII photochemical efficiency (*F*_v_/*F*_m_), PSII quantum yield (ΦPSII) and non-photochemical quenching (NPQ), between salinity treatments (**Figure 4**). These last three parameters remained unchanged in the salt-tolerant cultivar Sakha 93 until the mild salinity level, and a significant effect of salinity on these parameters only started to appear at the moderate salinity level. In contrast, the salt-sensitive cultivar Sakha 61 showed a significant decrease in *F*_v_/*F*_m_ and ΦPSII, and an increase in NPQ with increasing salinity levels (**Figure 4**).

### Ion Concentrations

**FIGURE 5:**
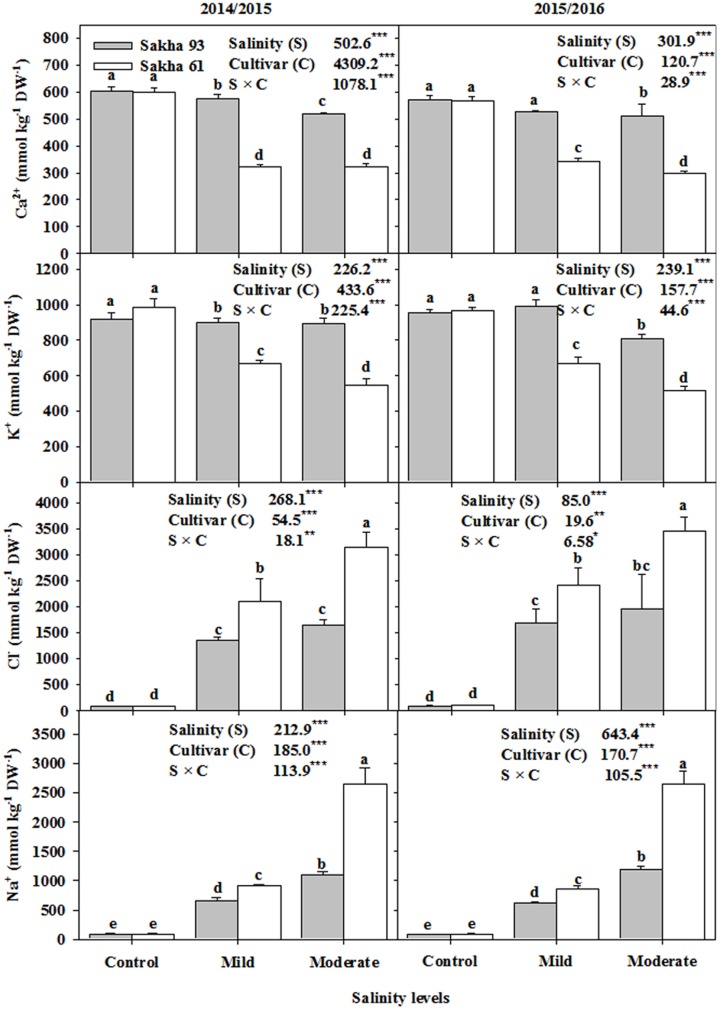
**Effects of different salinity levels on ion contents in leaves (Na^+^, Cl^-^, K^+^, Ca^2+^) at flowering stage of two wheat cultivars in 2014/2015 and 2015/2016**. Vertical bars indicate standard error (*n* = 3). Bars labeled with the different letters are significantly different at *P* < 0.05. The stars ^∗^, ^∗∗^ and ^∗∗∗^ indicate significant at 0.05, 0.01, and 0.001, respectively, according to *F*-values of ANOVA test.

**FIGURE 6:**
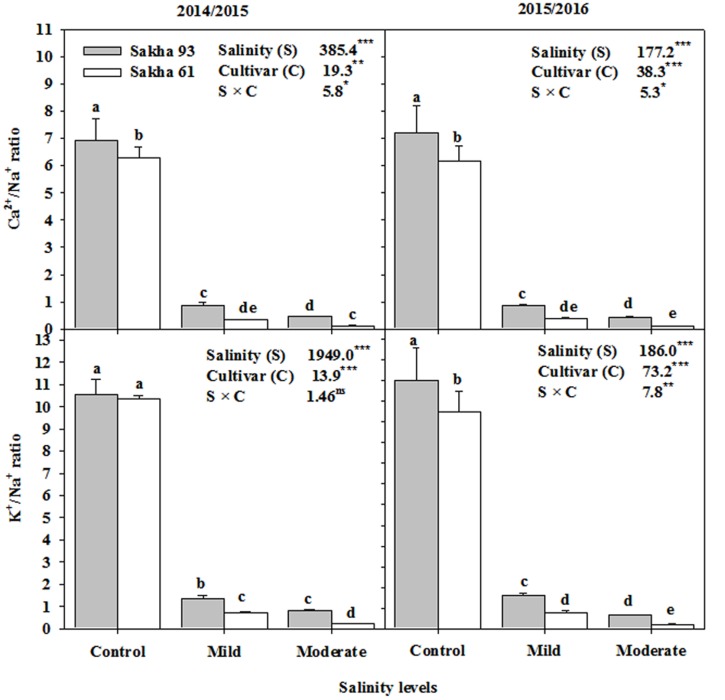
**Effects of different salinity levels on K^+^/Na^+^ and Ca^2+^/Na^+^ ratios at flowering stage of two wheat cultivars in 2014/2015 and 2015/2016**. Vertical bars indicate standard error (*n* = 3). Bars labeled with the different letters are significantly different at *P* < 0.05. The stars ^∗,^^∗∗^ and ^∗∗∗^ indicate significant at 0.05, 0.01, and 0.001, respectively, according to *F*-values of ANOVA test.

Based on the ANOVA analysis, the shoot Na^+^, Cl^-^, K^+^, and Ca^2+^ ion concentrations and the K^+^/Na^+^ and Ca^2+^/Na^+^ ratios were significantly different (*P* < 0.001 or 0.01) for the salinity treatments, cultivars, and S × C interactions (**Figures 5**, **6**). In general, as the salinity levels increased, the concentrations of toxic ions (Na^+^ and Cl^-^) also increased, while the concentrations of essential ions (K^+^, and Ca^2+^) decreased. However, the salt-tolerant cultivar Sakha 93 always exhibited good control of toxic ion accumulation and maintained higher essential ion concentrations under both the mild and moderate salinity levels than did the salt-sensitive cultivar Sakha 61 (**Figure 5**). Sakha 93 also maintained significantly higher shoot K^+^/Na^+^ and Ca^2+^/Na^+^ ratios than Sakha 61, even in the control treatment, with the exception of the K^+^/Na^+^ ratio in the first season (**Figure 6**).

### Principle Component Analysis (PCA)

**FIGURE 7:**
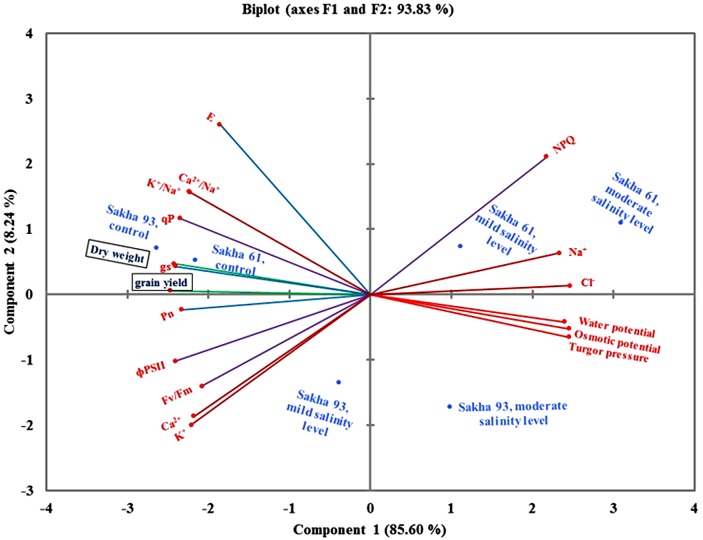
**Biplot of principle component analysis for the first two principle components of all parameters, cultivars, salinity levels and two growing seasons**. *F*_v_/*F*_m_, maximum quantum PSII photochemical efficiency; ΦPSII, quantum yield of PSII; qP, photochemical quenching coefficient; NPQ, non-photochemical quenching; Pn, net photosynthesis rate; gs, stomatal conductance; E, respiration rate.

The results of the PCA obtained from data of all agro-physiological parameters of the two cultivars subjected to different salinity levels are illustrated in **Figure 7**. The first two components explained 93.83% of the total variation between parameters. The first and second PCA explained 85.6 and 8.24% of the total variability, respectively. Interestingly, the leaf water relations parameters and toxic ion concentrations (Na^+^ and Cl^-^) were grouped together and revealed the identification of the salt-sensitive cultivar Sakha 61 in mild and moderate salinity levels, as well as the salt-tolerant cultivar Sakha 93 under the moderate salinity level. The two agronomical parameters were grouped with stomatal conductance, K^+^/Na^+^ ratio, and Ca^2+^/Na^+^ ratio and the angle between the vectors of these parameters was acute. Both cultivars were favored by these parameters under the control treatment. The salt-tolerant cultivar Sakha 93 was favored by two parameters of chlorophyll fluorescence (*F*_v_/*F*_m_ and ΦPSII) and two essential ion concentrations (K^+^ and Ca^2+^) (**Figure 7**).

## Discussion

Although considerable efforts have been made to identify screening criteria that best describe the salinity tolerance of wheat genotypes, the majority of experiments working on developing these criteria are carried out under controlled greenhouse conditions and using small pots or solution cultures as the growing media. Plants grown under such conditions are generally not exposed to situations predominantly found in natural saline field conditions, such as temporal and spatial variation in the physical and chemical properties of the soil, considerable fluctuation in salt concentrations and water content in the root zone growth medium, high variability in macro-environmental conditions (diurnal temperature and humidity), and highly complex interactions between macro- and micro-environmental conditions that surround the plants at different growth stages (Munns and James, 2003; El-Hendawy et al., 2009; Tavakkoli et al., 2010). Many of these complex conditions experienced by the plant in the field should be represented when plant breeders or physiologists attempt to verify the plant traits that can be used as screening criteria for salt tolerance. Therefore, the SWRT has been used in this study to simulate the majority of these factors, especially in the root zone.

### Quantifying the Effects of Salinity on Growth and Yield of Wheat Cultivars

Either under control or under saline conditions, morphological measurements of biomass and grain yield are frequently used as screening criteria for evaluating salt tolerance of wheat genotypes. This is because both of these parameters are functions of many physiological processes and integrate the response of these processes to salt stress at different growth stages and at the whole plant level (Munns and James, 2003; El-Hendawy et al., 2005a,b; Oyiga et al., 2016). In this study, SDW at the flowering stage and GYPH gradually declined with increasing salinity levels in both cultivars, but the decline was significantly lower in the salt-tolerant cultivar Sakha 93 than in the salt-sensitive cultivar Sakha 61 (**Figure 1**). This reduction in both parameters may be related to increasing NaCl concentrations in the root zone, which reduces the plants’ ability to take up sufficient water from the soil, in turn causing a water deficit within the plant, and/or an inability to regulate the net uptake of harmful ions, especially Na^+^ and Cl^-^, or essential ions such as K^+^ and Ca^2+^, in turn causing a specific ion toxicity and/or nutritional imbalance. However, the differential responses of both parameters to salinity stress observed between Sakha 93 and Sakha 61 cultivars indicate that the salt-tolerant cultivar might possess a more efficient salt tolerance mechanism than the salt-sensitive one. Such a mechanism would protect the plant from osmotic injury and the specific ion toxicity effects of salt stress. These mechanisms of salt tolerance based on various physiological traits will be discussed below in more detail, as well as the potential for exploiting these mechanisms as useful selection criteria for discriminating salt tolerance between the two wheat cultivars will also be discussed.

### Interpreting the Relationship between the Different Physiological Parameters

When plants grow under salt stress conditions, the physiological drought stress, which is a result of a difficulty in withdrawing water from the soil due to a decreased osmotic and matric potentials of the soil, results in a marked reduction in the leaf water potential (becomes more negative). This decrease in leaf water potential is usually accompanied by a significant reduction in the leaf osmotic potential through passive dehydration and/or active accumulation of organic and/or inorganic osmolytes (Pérez-López et al., 2009; Ashraf and Ashraf, 2015; Oyiga et al., 2016). Disrupting the balance between the leaf water potential and the leaf osmotic potential leads to dramatic changes in leaf turgor pressure, which is considered as the major factor, along with K^+^, in the control of stomatal opening. Since stomata are the main entrance points for CO_2_ uptake for photosynthesis and water evaporation from the leaves (transpiration), disturbed stomatal conductance leads to trouble in rates of photosynthesis and transpiration (Vysotskaya et al., 2010; Saqib et al., 2013). Because of this closed circuit between these physiological process, most parameters related to leaf water relations and photosynthesis have been routinely and effectively used as selection criteria for evaluating salt tolerance in a number of crops, such as wheat (Ashraf and Shahbaz, 2003; El-Hendawy et al., 2007; Munns and Tester, 2008; Saqib et al., 2013; Ashraf and Ashraf, 2015; Oyiga et al., 2016), barley and faba bean (Jiang et al., 2006; Tavakkoli et al., 2010; Vysotskaya et al., 2010), chickpea (Flowers et al., 2010), rice (Sanni et al., 2012), quinoa (Razzaghi et al., 2011), brassica (Singh et al., 2010), sunflower (Siddiqi and Ashraf, 2008), and cotton (Zhang et al., 2014). In this study, mild and moderate salinity levels induced a significant reduction in leaf water and osmotic potentials (the values of both parameters becoming more negative), stomatal conductance, photosynthesis rate, and transpiration rate. They also resulted in a higher leaf turgor pressure value than that found in the control treatment. However, the salt-tolerant cultivar Sakha 93 exhibited lower leaf water potential, leaf osmotic potential, leaf turgor pressure, and transpiration rate and higher stomatal conductance and photosynthesis rates than the salt-sensitive cultivar Sakha 61 (**Figures 2**, **3**), which reflected the better growth and productivity of Sakha 93 under salinity stress.

Less negative values of leaf water and osmotic potentials were exhibited by Sakha 93 than Sakha 61, demonstrating that this cultivar may possess a better ability to maintain a balance between water uptake and transpiration rate, as well as an effective strategy to adjust osmotic pressure through the accumulation of more adequate inorganic ions like K^+^ and Ca^2+^ and/or compatible organic substances, which are known to play an important role in the maintenance of leaf water potential and turgor pressure under salt stress (Pérez-López et al., 2009; Atiq-ur-Rahman et al., 2014). The salt-sensitive cultivar Sakha 61 also had the ability to maintain leaf turgor pressure, even though it exhibited a remarkable reduction in leaf water and osmotic potential when compared to Sakha 93 (**Figure 2**). These data may indicate that the increase in the transpiration rate in Sakha 61 (**Figure 3**), which is likely to be greater especially under open field conditions, may significantly contribute to increasing the inward water flow and subsequently leading to an increase in the flow of toxic ions (Na^+^ and Cl^-^) within the transpiration stream (Zheng et al., 2008; Vysotskaya et al., 2010). This can be deduced from the higher concentrations of Na^+^ and Cl^-^ in the shoot of Sakha 61 than in those of Sakha 93, which is addressed later. Because of the observed accumulation of toxic ions, it is reasonable to suggest that the osmotic adjustment in Sakha 61 is ensured mainly by the higher contribution of toxic ions (Na^+^ and Cl^-^). The results of leaf water relations parameters (leaf water potential, leaf osmotic potential, and turgor pressure) for the two cultivars suggest that the consideration of these parameters as screening criteria for evaluating salt tolerance under field conditions may depend on the machinery of the osmoregulatory processes that could be accomplished by toxic ion uptake in salt-sensitive genotypes. Therefore, if osmotic adjustment is indeed achieved purely by the increase in toxic ions, as suggested for Sakha 61, the leaf water relations, especially the leaf osmotic potential and leaf turgor pressure, should be a complementary by the other parameters, such as leaf injury and photosynthesis rate, when they are used as screening criteria under field conditions.

### Quantifying the Effects of Salinity on Photosynthesis

In this study, the salt-sensitive cultivar Sakha 61 showed a higher reduction in photosynthesis rate than the tolerant cultivar Sakha 93 under mild and moderate salinity levels (**Figure 3**). This suggests that the accumulation of toxic ions in leaves may be one factor inhibiting photosynthesis in Sakha 61. In addition, the stomatal factor may also be the other reason for the observed inhibition of photosynthesis under salt stress (El-Hendawy et al., 2005a; Nishimura et al., 2011; Ahmed et al., 2013; Zhang et al., 2014). In this study, the stomatal conductance of Sakha 61 significantly decreased with salinity levels increasing from mild to moderate, whereas there was no significant difference in this parameter in Sakha 93 (**Figure 3**). A significant reduction in stomatal conductance in Sakha 61 may be a result of elevated levels of toxic ions, especially if these toxic ions are not segregated in the vacuole, and/or there is a lack the availability of potassium in guard cells, which induces a significant reduction in turgidity of these cells and a disruption of normal stomatal functions (Burman et al., 2003; Najafi et al., 2007). Therefore, based on the clear differences in photosynthesis rate and stomatal conductance between the salt-tolerant and salt-sensitive cultivars, it may be inferred that both parameters could be considered as useful screening criteria and sufficient for discriminating salt tolerance among genotypes of wheat under field conditions.

### Chlorophyll Fluorescence Parameters as an Effective and Rapid Screening Tool for Salt Tolerance

Interestingly, the negative impacts of salt stress on photosynthetic performance can be detected even under mild salinity conditions or short-term salinity stress before the irreversible morphological damage becomes visible (Zarco-Tejada et al., 2003). It is possible to detect these negative impacts by measuring different chlorophyll fluorescence parameters, which are sensitive to any perturbation that directly affects the plant metabolism and photosynthesis apparatus (Baker and Rosenqvist, 2004; Murchie and Lawson, 2013). In this study, the two tested cultivars clearly showed significant variation in all chlorophyll fluorescence parameters, except for photochemical quenching (qP) (**Figure 4**), which makes these fluorescence parameters an effective and rapid screening tool for distinguishing between salt-tolerant and salt-sensitive genotypes of wheat.

Sakha 61 exhibited a much lower *F*_v_/*F*_m_ value under moderate salinity levels (averaged across two seasons, the value decreased to 0.56) than the standard value (standard value of this parameter under non-stress conditions ranged from 0.80 to 0.86 in C_3_ plants). However, the value of this parameter in Sakha 61 under mild salinity level and the value exhibited by Sakha 93 under mild and moderate salinity levels, were comparable to the standard value (**Figure 4**). This result indicates that the suitability of this parameter for screening genotypes for salt tolerance depends on the salinity level and the level of salt tolerance of the genotypes.

The close relationship between the PSII quantum yield (ΦPSII), which is used to estimate the rate of electron transport (ATP and NADPH) through PSII, and the CO_2_ assimilation rates makes this parameter an important criterion for evaluating genotypes under types of different environmental stress (Baker and Rosenqvist, 2004; Murchie and Lawson, 2013). The results of the present study confirm that the ΦPSII can be successfully used to differentiate between salt-sensitive and salt-tolerant cultivars even under mild salinity levels. The reduction in this parameter due to salt stress was higher in the salt-sensitive cultivar (**Figure 4**). The decrease in ΦPSII exhibited by the salt-sensitive cultivar is most likely related to the significant decrease in stomatal conductance shown by this cultivar under salinity treatments, as discussed before. The limitation imposed by a decrease in stomatal conductance is often accompanied by decreases in the efficiency of electron transportation, which could result in decreases in the ATP and NADPH consumption in the photosynthetic metabolism and, consequently, in ΦPSII. Meanwhile, the ability of the salt-tolerant cultivar Sakha 93 to maintain stomatal conductance and to keep low concentrations of toxic ions in leaves under salt stress could maintain a higher capacity of ΦPSII than did Sakha 61.

It is noteworthy that the lower PSII quantum yield (ΦPSII) was obviously associated with a significant increase in non-photochemical quenching (NPQ) in the salt-sensitive cultivar Sakha 61, even under mild salinity level (**Figure 4**). This increase could be related to an enhancement in heat dissipation of excitation energy in the PSII antenna system, which serves as a way to maintain a proper balance between the decrease in photosynthesis and linear photosynthetic electron transport in order to avoid photodamage in PSII (Qiu et al., 2003; Li et al., 2010). As discussed above, there is no doubt that the close relationship between stomatal conductance, photosynthesis rate, PSII (ΦPSII) and NPQ means that these parameters can serve as effective and rapid screening criteria for discriminating salt tolerance between wheat genotypes.

### Exclusion and Selectivity of Ions as a Key Physiological Mechanism Contributing to Wheat Salt Tolerance

The ability of plants to maintain ion homeostasis under saline conditions is still considered a reliable indicator and an effective mechanism for salt tolerance. Most studies report that high external NaCl concentrations result in intense competition between ions for absorption at the site of ion uptake, especially between Na^+^ and K^+^ ions, due to the similarity in the physio-chemical properties of both ions, which does not act in favor of metabolic functions essential for adaptation to salt stress (Kaya et al., 2007; Rasheed et al., 2014; Ashraf and Ashraf, 2015). Therefore, salt tolerance in most genotypes coincides with higher affinity for K^+^ over Na^+^ in ion uptake. In this study, ion analysis revealed that the salt-tolerant cultivar Sakha 93 accumulated less Na^+^ and Cl^-^ than Sakha 61 under mild and moderate salinity conditions. An opposite trend was noted for K^+^ and Ca^2+^ accumulation (**Figure 5**). The lower concentrations of Na^+^ and Cl^-^ in Sakha 93 indicate that this salt-tolerant cultivar has a superior ability to exclude Na^+^ (1039.9 versus 1395.5 mmol kg^-1^ DW, and 2163.0 versus 3300.1 mmol kg^-1^ DW, averaged over two seasons) and Cl^-^ (1642.6 versus 2269.8 mmol kg^-1^ DW, and 2257.7 versus 3302.2 mmol kg^-1^ DW as averaged over two seasons) from shoots than the salt-sensitive cultivar Sakha 61, when grown under mild and moderate salinity levels, respectively (**Figure 5**). Therefore, the exclusion of Na^+^ and Cl^-^, which minimized the amount of both ions accumulating in the metabolically active areas of cells, appeared to significantly contribute to the salt tolerance exhibited by Sakha 93.

Sakha 93, which showed a stronger ability to exclude Na^+^ and Cl^-^, also exhibited higher K^+^ and Ca^2+^ concentrations, and higher K^+^/Na^+^ and Ca^2+^/Na^+^ ratios than Sakha 61 (**Figure 6**). This indicates that salt tolerance is not only related to the ability to restrict Na^+^ transport to the shoot, but also to the affinity for K^+^ and/or Ca^2+^ over Na^+^ (Kaya et al., 2007; Kausar et al., 2014; Oyiga et al., 2016). Although the mechanism of Na^+^ exclusion could protect the plant from specific-ion toxicity, the low Na^+^ uptake should be associated with high uptake of K^+^ and Ca^2+^, because sufficient levels of these ions play an ideal role in osmotic adjustment, without the energy cost incurred during the synthesis of compatible organic substances (Munns and Tester, 2008; Tavakkoli et al., 2010). Therefore, selective uptake of K^+^ and/or Ca^2+^ over Na^+^ cannot be ruled out as a key physiological mechanism contributing to wheat salt tolerance under field conditions, because the plants must cope with Na^+^ toxicity, simultaneously maintaining adequate osmotic adjustment.

### Assessment of Salt Tolerance Parameters Using Principal Component Analysis

**Figure 7** provides a comprehensive picture of the inter-relationships between all measured parameters. It explains which parameters can be used as individual, interchangeable, or supplementary screening criteria for evaluating salt tolerance under simulated field conditions. These interrelationships between parameters are usually examined using the angles between the parameter vectors on the biplot of the principal component analyses (PCAs). An acute angle indicates close and strong correlations, and vice versa with the obtuse angle. A right angle indicates no correlation between parameters, while a straight angle reflects a negative correlation between them. Based on these principles, the leaf water relations parameters (water potential, osmotic potential, and turgor pressure) and the toxic ion concentrations (Na^+^ and Cl^-^) were grouped together. This suggested that these parameters had a close relationship between them and could be used as complementary screening criteria for salt tolerance, because osmotic adjustment could be ensured mainly by the higher accumulation of toxic ions, as demonstrated in the salt-sensitive cultivar Sakha 61. The straight angle between the toxic ions and the agronomic parameters (dry weight and grain yield), essential ion concentrations (K^+^ and Ca^2+^), and photosynthetic parameters (Pn, and gs) suggests that the exclusion of toxic ions is still an important mechanism in salinity tolerance. The genotypes with lower toxic ion concentrations have greater biomass and yield, and are able to protect their photosynthesis apparatus under saline field conditions. Therefore, the agronomic parameters, concentrations of essential ions, K^+^/Na^+^ ratio, or photosynthesis parameters can be used as individual or interchangeable screening criteria for salt tolerance. The straight angle of NPQ with *F*_v_/*F*_m_ and ΦPSII suggests that these three parameters can be used as alternative screening criteria for salt tolerance.

## Conclusion

Data gathered in this study indicate that the SWRT technique can be used as simulate field conditions to accurately appraise the salt tolerance of wheat genotypes. This work has given further weight for the use of some agro-physiological parameters as screening criteria for discriminating salt tolerance among wheat genotypes. Principal component analyses have provided a comprehensive picture of the interrelationships between all analyzed parameters and indicated that some physiological parameters can be used individually, while others are interchangeable with other parameters when they are used as screening criteria for evaluating the salt tolerance of wheat genotypes under field conditions.

## Author Contributions

SE-H performed the experiments, analyzed the data, and edited the manuscript, WH, YR, and KA designed the experiment, followed upon data collection, provided support through the PCA statistical analysis, SE-H, NA-S final approval of the version to be published.

## Conflict of Interest Statement

The authors declare that the research was conducted in the absence of any commercial or financial relationships that could be construed as a potential conflict of interest.
